# Elevated serum lactate as a predictor of outcomes in patients following major abdominal surgery at a tertiary hospital in Uganda

**DOI:** 10.1186/s12893-021-01315-y

**Published:** 2021-08-07

**Authors:** Kiyemba Henry, Kadondi Merab, Muyanja Leonard, Kintu-Luwaga Ronald, Kakembo Nasser, Galukande Moses

**Affiliations:** 1 Department of Surgery, College of Health Sciences, Makerere University, Kampala, Uganda; 2 Department of Surgery, Mulago National Referral Hospital, Kampala, Uganda

**Keywords:** Elevated serum lactate, Major abdominal surgery, Outcome, Post-operative complications, Type of surgery

## Abstract

**Introduction:**

Major abdominal surgery is still a great contributor to postoperative morbidity and mortality in developing countries. Major abdominal surgery leads to hypoperfusion, which has an impact on postoperative morbidity and mortality. Lactate, a biomarker for hypoperfusion is under utilized in Uganda. The study aimed to investigate the association between elevated serum lactate and outcomes (in-hospital mortality, SSI and length of hospital stay) in patients following major abdominal surgery.

**Methods:**

A prospective observational cohort study was done with 246 eligible patients recruited. Stratified sampling was carried out till desired sample size was achieved. Demographic and perioperative data were collected, serum lactate levels were measured at induction and immediately after surgery with serial measurements being done after 12, 24 h post operatively. Participants were followed up to assess outcomes. Data analysis was done using STATA version 14.0.

**Results:**

A total of 130 patients (52.8%) had elevated serum lactate levels. Elevated serum lactate predicted in-hospital mortality and surgical site infection. The accuracy of elevated serum lactate to predict mortality with AUROC of 0.7898 was exhibited by the 24 h lactate values. Elevated serum lactate predicted surgical site infection accurately with AUROC 0.6432. Length of hospital is strongly associated with elevated serum lactate with p-value of 0.043. Patients with elevated serum lactate on average have a longer length of hospital stay at 5.34 ± 0.69.

**Conclusion:**

Elevated serum lactate was associated with in-hospital mortality, surgical site infection and longer length of hospital stay. Serum lactate levels done at 24 h were most predictive of mortality and surgical site infection.

## Introduction

Major abdominal surgeries are performed in their millions around the world [1]. In Africa, about 143 million surgeries occur annually [2]. In East Africa, 45 operations are performed per 10,000 people [3]. Advancements in monitoring and perioperative patient care have reduced majority of the burden of postoperative complications and mortality. However, the greater percentage of the remaining burden is still present in the developing world. Mortality and morbidity are greatly dependent on vascular flow post operatively and compromise to this results in a wide array of outcomes [4].

Lactate has excellent sensitivity for assessing cellular damage especially from tissue hypoperfusion [5]. Elevated serum lactate or hyperlactatemia is associated with increasing rates of mortality [6, 7] and this necessitates prompt identification of the patients and discontinuation of the offending agent. Hyperlactatemia has an association with morbidity and Post Operative Complications (POCs) following major abdominal surgery [6–8].

Monitoring of lactate is widely used in critically ill patients such as those in Intensive Care Units (ICUs) and in patients following cardiothoracic surgery [9]. The significance of lactate monitoring in patients following major abdominal surgery, however, is still questionable especially in developing countries and there is still a deficiency in accurately and inexpensively detecting hypoperfusion in surgical patients more so following major abdominal surgery. Lactate is a potential predictor of this but is still under-utilized.

Therefore the purpose of the study was to investigate the association between hyperlactatemia and outcomes in patients following major abdominal surgery at Mulago National Referra Hospital.

## Methods and materials

Prospective observational cohort study conducted at Mulago National Referral Hospital (MNRH), a public tertiary facility in Kampala, the administrative capital city of Uganda. The study population included all patients admitted to MNRH during the study period to undergo major abdominal surgery that had a diagnosis that led to major abdominal surgery who consented to participate in the study. We excluded diabetic patients on metformin from the study.

We used a sample size of 246 participants for the study with stratified sampling used and generation of sub groups as adults (> 18 years and above) and children (17 years and below). The proportions were in a ratio of 1:1 and consecutive sampling used within the subgroups till we acquired the desired sample size. The study variables included independent variable as hyperlactatemia: elevated serum lactate between 2 and 4 mmol/L just before surgery (L_0_), immediately after surgery (L_1_), after 12 h (L_2_), after 24 h (L_3_); dependent variables included: In-hospital mortality: death that occurred within hospital, Surgical Site Infection: infection that occur at operative wound following a surgical procedure, Length of hospital stay: time from admission till discharge or when outcome of interest was obtained. Other variables; comorbidity, time interval between diagnosis and surgical intervention, onset of symptoms, age, type of surgery, duration of surgery, treatment given, ASA status, organs/structures involved.

We used a data collection form to acquire socio-demographic information and intra operative variables.

An initial serum lactate level at induction was obtained using a lactate meter TD-4261A—a point of care testing system for bed side testing of lactate. Blood samples for serum lactate were then collected immediately after surgery and subsequently at 12 h and 24 h following the surgery. The patients were assessed for surgical site infection and followed up till outcome of interest was achieved or up to a maximum of 30 days into the post-operative period if still admitted.

### Data analysis

We entered the data into Epidata version 3.1 and subsequently analysed with STATA version 14.0 using descriptive and analytical statistics. We summarized all continuous variables following a normal distribution using means and standard deviations. Those not normally distributed were summarized using median and inter-quartile ranges. Categorical data was summarized using proportions and percentages.

We used the logistic regression to determine associations between hyperlactatemia and in-hospital mortality, Surgical site infection (SSI) and linear regression to determine association between hyperlactatemia and length of hospital stay through calculation of odds ratios (OR) and their corresponding 95% confidence intervals (CI).

Independent variables significant at bivariate analysis at p-value less than 0.2 had multivariate analysis done. In multivariate analysis, we did a step wise backward method to identify significant independent variables at p-value less than 0.05. Serum lactate was assessed for interaction and confounding with the other independent variables.

The final logistic models predicting in-hospital mortality and surgical site infection had testing for goodness of fit by determining the sensitivities and specificities of lactate in predicting the outcomes. To assess accuracy we constructed Receiver Operator Curves (ROCs) and generated Area Under Receiver Operator Curves (AUROC).

### Ethical considerations

We obtained approval to carry out the study from the Department of Surgery, School of Medicine Research and Ethics Committee (reference number #REC REF 2019-094). Each study participant had written informed consent done before enrollment into the study and confidentiality ensured through the use of identification numbers. Informed consent was obtained from a parent or legal guardian for a participant under 18 years of age.

## Results

A total of 246 patients under major abdominal surgery and were evaluated for the purpose of this study. Patients characteristics are summarized in Table 1.

**Table 1:** Characteristics of 246 patients who underwent major abdominal surgery

Characteristic (n = 246)	Frequency	Percentage
Age		
< 18 years	121	49.2
≥ 18 years	125	50.8
Gender		
Male	129	52.4
Female	117	47.6
Type of surgery		
Elective	62	25.2
Emergency	184	74.8
ASA status		
I	50	20.3
II	110	44.8
III	82	33.3
IV	4	1.6
Duration between diagnosis and surgery for emergencies (days), n = 184		
Within 1 day	119	64.7
Longer than a day	65	35.3
Duration of surgery (hours)		
** ≤ 1.5**	180	73.2
** > 1.5**	66	26.8
Pre-surgery treatment		
Yes	52	21.1
No	194	78.9
Intra operative treatment (litres)		
≤ 1	94	38.2
> 1	152	61.8
Comorbidities		
No	183	74.4
Yes	63	25.6
Organs involved		
One organ	192	78.0
More than one organ	54	22.0
Serum lactate		
Normal (≤ 2.0 mmol/L)	116	47.2
High (> 2.0 mmol/L)	130	52.8

The bold was indicative of the statistically significant **p** values of different variables

This study observed the average lactate values between age groups were 3.18 ± 0.44 for patients less than 18 years and 2.74 ± 0.24 for those above 18 years

### Outcomes

#### In hospital mortality

This study observed that 34 patients died (13.8%). Our study showed that elevated serum lactate was strongly associated with mortality (Table 2). Table 4 shows the prognostic value of serum lactate in predicting in-hospital mortality. Statistically, the following factors increased the risk of mortality; patients with comorbidities, duration of surgery ≥ 1.5 h, delay between diagnosis and surgery greater than a day and finally age > 18 years as seen in Table 2.

**Table 2:** Factors associated with in-hospital mortality among patients who underwent major abdominal surgery at MNRH during the study period

Characteristic (n = 246)	Univariate			Multivariate		
	cOR	95% CI	p-value	aOR	95%CI	p-value
Age						
< 18 years	1					
≥ 18 years	0.30	0.21–0.43	< 0.001	0.24	0.06–0.90	**0.034**
Gender						
Male	1					
Female	0.23	0.15–0.34	< 0.001	0.19	0.06–0.64	**0.007**
Type of surgery						
Elective	1					
Emergency	2.67	1.98–3.60	< 0.001	2.27	0.08–2.33	0.331
ASA status						
I	1					
II	2.98	1.09–3.87	0.020	1.20	0.45–3.22	0.710
III	3.68	1.76–5.65	0.001	2.06	0.74–4.96	0.078
IV	6.13	2.89–9.97	< 0.001	2.39	0.13–4.18	0.462
Duration between diagnosis & surgery for emergencies (days)						
Within 1 day	1					
Longer than a day	1.77	1.59–2.90	0.043	1.45	1.13–1.86	**0.003**
Duration of surgery (hours)						
≤ 1.5	1					
> 1.5	2.63	1.75–4.00	< 0.001	3.17	1.01–9.98	**0.049**
Pre-surgery treatment						
Yes	1					
No	2.09	1.54–4.03	< 0.001	1.78	0.29–11.11	0.531
Intraoperative treatment (litres)						
≤ 1	1					
> 1	0.30	0.19–0.47	< 0.001	1.51	0.12–2.69	0.753
Comorbidities						
No	1					
Yes	1.15	1.10–1.19	< 0.001	1.23	1.11–1.36	** < 0.001**
Organs involved						
One	1					
More than one organ	1.34	1.20–1.50	< 0.001	1.06	0.71–1.25	0.675
Serum lactate						
Normal (≤ 2.0 mmol/L)	1					
High (> 2.0 mmol/L)	3.79	2.09–6.84	< 0.001	5.20	1.51–17.92	**0.009**

The bold was indicative of the statistically significant **p** values of different variables

Mortality n = 34, 13.8%

**Fig. 1:**
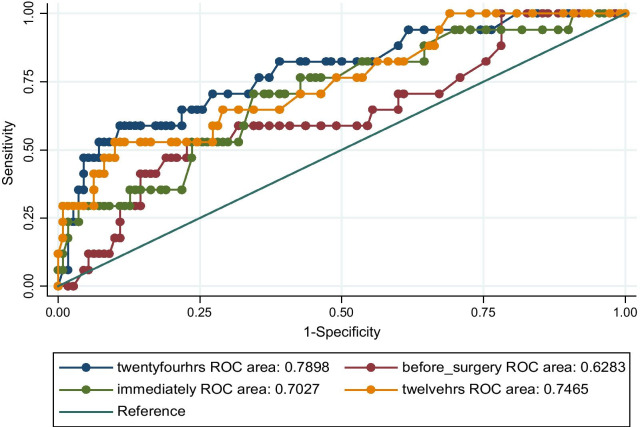
ROC of serum lactate levels at four time points following major abdominal surgery in predicting mortality. 24 h serum lactate levels were the highest in predicting mortality

The ROC analysis (Fig. 1) demonstrated that elevated serum lactate was accurate in predicting in-hospital mortality and that the 24 h serum lactate levels had the best AUROC of 0.7898 which corresponds to good accuracy.

#### Surgical site infection

**Fig. 2:**
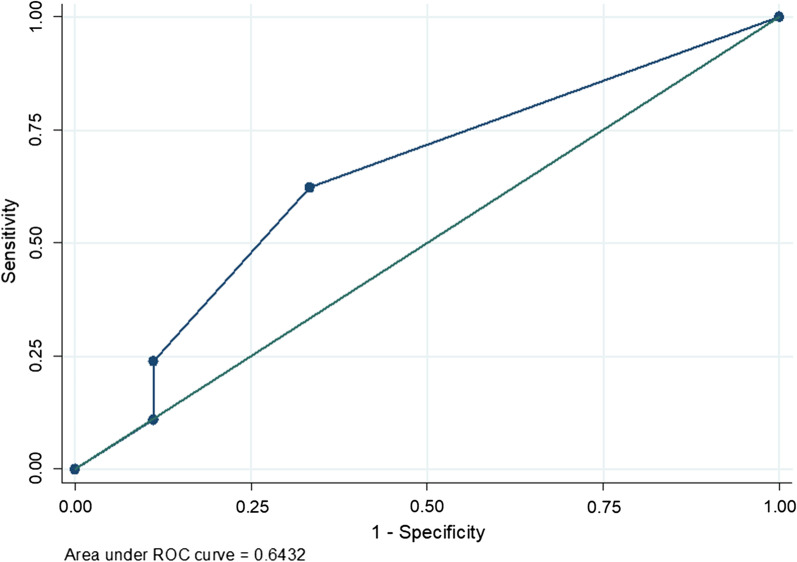
ROC of 24 h serum lactate levels following major abdominal surgery to predict surgical site infection. AUROC was 0.6432

In this study, 14.6% of the patients developed surgical site infections. Significant factors included elevated serum lactate and lack of pre surgical treatment (Table 3). Elevated serum lactate with the prognostic value showed in Table 4 and an accuracy of 0.6432 AUROC (Fig. 2).

**Table 3:** Prognostic value of serum lactate for in-hospital mortality and SSIs

Characteristic (serum lactate for)	Sensitivity (%)	Specificity (%)	Positive predictive value (%)	Negative predictive value (%)
In-hospital mortality	35.29	96.26	60.00	90.35
SSIs	94.40	54.30	26.20	98.20

**Table 4:** Factors associated with surgical site infection among patients who underwent major abdominal surgery at MNRH during the study period

Characteristic	Univariate			Multivariate		
	cOR	95% CI	p-value	aOR	95% CI	p-value
Age						
< 18 years	1					
≥ 18 years	2.70	1.94–3.75	< 0.001	1.41	0.58–3.45	0.455
Type of surgery						
Elective	1					
Emergency	2.51	1.88–3.36	< 0.001	1.33	0.55–3.21	0.521
ASA status						
I	1					
II	0.78	0.44–1.26	0.675	0.79	0.74–2.14	0.389
III	1.42	0.95–4.55	0.080	1.10	0.23–1.98	0.078
IV	3.64	1.59–7.65	0.042	2.60	0.81–3.78	0.090
Duration between diagnosis and surgery (days)						
Within 1 day	1					
Longer than a day	3.23	2.05–5.07	< 0.001	1.69	0.57–4.99	0.345
Duration of surgery (hours)						
≤ 1.5	1					
> 1.5	2.98	1.93–4.60	< 0.001	1.63	0.63–4.35	0.307
Pre-surgery treatment						
Yes	1					
No	4.65	2.91–7.44	< 0.001	6.20	1.12–4.26	**0.036**
Intra operative care (litres)						
≤ 1	1					
> 1	0.37	0.25–0.58	< 0.001	0.81	0.18–3.58	0.780
Comorbidities						
No	1					
Yes	1.13	1.09–1.17	< 0.001	1.01	0.84 -1.22	0.894
Organs involved						
One organ	1					
More than one organ	3.35	1.16–5.46	< 0.001	1.02	0.81–1.28	0.873
Serum lactate						
Normal (≤ 2.0 mmol/L)	1					
High (> 2.0 mmol/L)	2.87	1.99–4.13	< 0.001	1.25	1.11–3.70	**0.008**

The bold was indicative of the statistically significant **p** values of different variables

SSI n = 36; 14.6%

#### Length of hospital stay

Length of hospital stay was associated to elevated serum lactate (Table 5). ASA III (p-value 0.003) and duration of surgery > 1.5 h (p-value 0.032) were significantly associated with prolonged stay. Those patients with elevated serum lactate on average had a longer length of hospital stay as compared to those that had normal lactate levels (Table 6).

**Table 5:** Factors associated with length of hospital stay among patients who underwent major abdominal surgery at MNRH during the study period

Characteristic	Univariate			Multivariate		
	cOR	95% CI	p-value	aOR	95% CI	p-value
Age						
< 18 years	1					
≥ 18 years	5.11	2.40–6.49	< 0.001	3.47	0.08–0.66	0.390
Gender						
Male	1					
Female	3.27	2.64–3.90	< 0.001	2.03	0.63–6.62	0.237
Type of surgery						
Elective	1					
Emergency	2.82	2.33–3.31	< 0.001	2.64	0.64–3.20	0.737
ASA status						
I	1					
II	4.12	2.76–5.49	< 0.001	2.22	0.43–5.67	0.316
III	7.41	5.82–8.99	< 0.001	6.28	2.74–8.97	**0.003**
IV	3.00	4.27–10.27	0.416	1.86	0.20–2.35	0.960
Duration between diagnosis & treatment for emergencies (days)						
Within 1 day	1					
Longer than a day	3.67	3.00–4.34	< 0.001	2.54	0.08–3.16	0.508
Duration of surgery (hours)						
≤ 1.5	1					
> 1.5	3.45	2.96–3.93	< 0.001	2.03	1.91–3.15	**0.032**
Pre-surgery treatment						
Yes	1					
No	3.81	3.08–4.53	< 0.001	1.13	0.60–5.86	0.637
Comorbidities						
No	1					
Yes	2.35	1.29–4.42	< 0.001	1.90	0.27–2.12	0.446
Organs involved						
One organ	1					
More than one organ	0.75	0.60–0.89	< 0.001	0.24	0.16–2.32	0.800
Serum lactate						
Normal (≤ 2.0 mmol/L)	1					
High (> 2.0 mmol/L)	5.34	3.86–6.82	< 0.001	1.75	1.43–2.37	**0.043**

The bold was indicative of the statistically significant **p** values of different variables

**Table 6:** Average length of hospital stay over serum lactate levels

Lactate/hospital stay	Mean in days	SD	95% CI
≤ 2 mmol/L	5.12	0.55	4.04–6.20
> 2 mmol/L	5.34	0.69	3.98–6.71

## Discussion

We set out to investigate the association between elevated serum lactate and of outcomes in patients following major abdominal surgery at MNRH.

### Patient characteristics

We found the age and gender profile similar to a study done in this setting by Kitara et al. [10] with majority of the patients being above 18 years of age and males.

We did not find comorbidity in 74.4% of the patients similar to a study done by Kitara et al., which had 78.9% of patients with no comorbidity [10]. In our study, this minimized the risk of confounding by comorbidities that could result in elevated serum lactate levels as stipulated by Lars et al. [11].

Our study had most surgeries as emergency cases (74.8%) consistent with a study by Kitara et al. [10]. For the emergency cases, when diagnosis was made, had surgery done within a day of diagnosis and these were 64.7% of all patients. About 78.9% of the patients requiring surgery had received no treatment prior to surgery. These can be attributed to logistical issues. However intra-operatively, 61.8% of the patients received more than 1 L of intravenous fluids as part of fluid therapy.

### Elevated serum lactate

Of the 246 patients recruited in the study, 130 patients (52.8%) had elevated serum lactate levels. A similar study had reference range for elevated serum lactate between 2 and 4 mmol/L [8].

The average lactate values between age groups were 3.18 ± 0.44 for patients less than 18 years and 2.74 ± 0.24 for those above 18 years. The difference in average values between the age groups was minimal and this reflects the ability of serum lactate to in-discriminatively predict hypoperfusion across various ages.

### Outcomes

#### In-hospital mortality

This study observed that 34 patients died (13.8%), a mortality rate similar to that found in a study done in MNRH by Kitara et al. who found a moratlity rate of about 14.5% [10]. Our study showed that elevated serum lactate was strongly associated with mortality with aOR 5.2 95%CI 1.51–17.92, p-value 0.009 which is in agreement with what Velickovic et al. observed in a study done in Europe and Okello et al. observed in Uganda at MNRH [8, 12]. Elevated serum lactate had a sensitivity 35.29%, specificity 96.26%, PPV 60%, NPV 90.35% proving it is a reliable biomarker, in agreement with other studies [8, 12, 13].

The ROC analysis demonstrated that elevated serum lactate was accurate in predicting in-hospital mortality and that the 24 h serum lactate levels had the best AUROC of 0.7898 which corresponds to good accuracy. These findings were similar to those in studies that were done in MNRH in trauma patients [12] and one done in Europe in elective patients post major abdominal surgery that had AUROC of 0.821 [8].

Statistically, the following factors increased the risk of mortality; patients with comorbidities p-value < 0.001, especially in the setting of hypoperfusion, similar to what was observed by Lars et al. [11]. The duration of surgery ≥ 1.5 h p-value 0.049 which could be attributed to the duration of tissue manipulation and as such compounding the hypoperfusion of the patients and result in elevation of serum lactate levels. The delay between diagnosis and surgery greater than a day had a p-value 0.003 resulting in delay to remove the insult that could be causing the hypoperfusion though this is usually attributed to logistical delays and finally age > 18 years usually corresponding to more critically ill patients had a p-value of 0.034 similar to what other studies noted [8, 12].

#### Surgical site infection

In this study, 14.6% of the patients developed surgical site infections. These findings were similar to rate of 14.1% that was observed in MNRH [10].

Consistent with studies by Velickovic et al., Cobianchi et al. and Bakker et al. [8, 14, 15], it was observed that elevated serum lactate was significantly associated with surgical site infection with an aOR 1.25 95%CI 1.11–3.72, p-value 0.008. Elevated serum lactate had a sensitivity of 94.4%, specificity of 54.3% and the accuracy of predicting SSI being sufficient at 0.6432 AUROC. In this study the accuracy for predicting SSI was sufficient in comparison to a study done in Europe [8], where the AUROC was 0.787. The difference in accuracy could be attributed to difference in geographical location of the two studies, which brings into consideration different antibiotic guidelines as well as pathogens responsible for SSI.

Patients not receiving pre-operative antibiotics, potentiating the risk of developing surgical site infection, would explain why lack of pre-surgery treatment was a significant factor with p-value of 0.036 as observed in our study.

#### Length of hospital stay

Length of hospital stay was associated withsurgical site infection and this was in keeping with a study by Seni et al. done at MNRH that looked at antimicrobial resistance in hospitalised surgical patients [16].

Patients with elevated serum lactate on average had a longer length of hospital stay at 5.34 ± 0.69 as compared to those that had normal lactate levels. Elevated serum lactate was associated with a longer length of hospital stay, with a p value of 0.043, consistent with other studies [7, 8, 17] though their patients had a longer length of stay compared to those in our study. This could be attributed to the volume of patients admitted to MNRH versus availability of space for admission of patients and therefore high turnover of patients.

In our study, patients that had duration of surgery > 1.5 h and ASA III were more likely to have longer duration of hospital stay with significant p-value of < 0.05. This can be explained by these patients being were more critically ill, having had longer periods of time of tissue manipulation intraoperatively and more severe forms of comorbidity present.

## Limitations

The study was carried out at tertiary health facility receiving referrals which could have caused random error especially at the time of sampling. Logistical delays that were out of our control especially erratic supply of necessities for surgery.

## Conclusion

Elevated serum lactate was associated with in-hospital mortality, surgical site infection and longer length of hospital stay. Serum lactate levels done at 24 h were most predictive mortality and surgical site infection compared to the other perioperative serum lactate levels.

## Data Availability

Data sets are available by friendly request to the corresponding author.

## Abbreviations

ASA: American Association of Anaesthesiologists
ASOS: African Surgical Outcome Study
AUROC: Area Under Receiver Operator Curve
MNRH: Mulago National Referral Hospital
POCs: Post Operative Complications
SSC: Surviving Sepsis Campaign
SSI: Surgical Site Infection
